# Schmallenberg Disease—A Newly Emerged Culicoides-Borne Viral Disease of Ruminants

**DOI:** 10.3390/v11111065

**Published:** 2019-11-15

**Authors:** Abaineh D. Endalew, Bonto Faburay, William C. Wilson, Juergen A. Richt

**Affiliations:** 1 Department of Diagnostic Medicine/Pathobiology, College of Veterinary Medicine, Kansas State University, Manhattan, KS 66506, USA; adendale@vet.k-state.edu (A.D.E.); bfaburay@vet.k-state.edu (B.F.); 2 United States Department of Agriculture, Agricultural Research Service, Arthropod-Borne Animal Disease Research Unit, Manhattan, KS 66506, USA

**Keywords:** Schmallenberg virus, Culicoides, ruminants

## Abstract

First appearing in 2011 in Northern Europe, Schmallenberg virus (SBV), an *Orthobunyavirus* of the Simbu serogroup, is associated with clinical disease mainly in ruminants such as cattle, sheep and goats. The clinical signs are characterized by abortion and congenital deformities in newborns. The virus is transmitted by Culicoides midges of the Obsoletus complex. SBV infection induces a solid protective immunity that persists for at least 4 or 6 years in sheep and cattle, respectively. SBV infection can be diagnosed directly by real-time RT-qPCR and virus isolation or indirectly by serological assays. Three vaccines are commercially available in Europe. This article provides a comprehensive literature review on this emerging disease regarding pathogenesis, transmission, diagnosis, control and prevention. This review also highlights that although much has been learned since SBV’s first emergence, there are still areas that require further study to devise better mitigation strategies.

## 1. Discovery and Genomic Structure of the Virus

In the fall of 2011, a new cattle disease was reported in Germany and The Netherlands associated with a drop in milk production, hyperthermia and diarrhea. Using a metagenomic approach on blood samples collected from clinically sick dairy cows, the causative agent was identified as a novel RNA virus [1]. Consequently, the agent was named Schmallenberg virus (SBV) after the locality in Germany where the outbreak occurred. 

**Figure 1:**
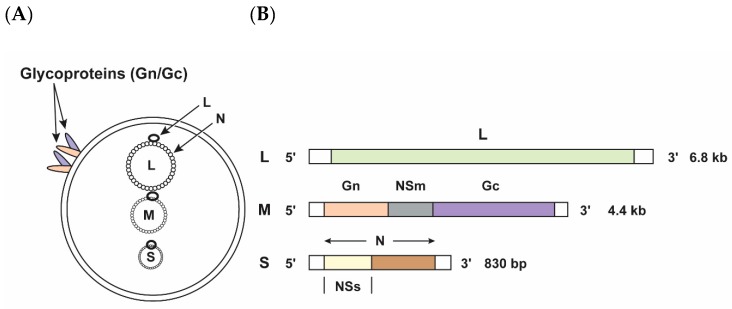
Schmallenberg virus (SBV) virion and genome organization. (**A**) The SBV large (L), medium (M), and small (S) segments are present in the virion and encapsulated with an envelope lipid bilayer containing the surface glycoproteins Gn and Gc. (**B**) Schematic representation of the SBV segments and the SBV coding strategy: L-segment: L, RNA-dependent RNA polymerase protein; M-segment: Gn and Gc, glycoproteins Gn and Gc; NSm, non-structural protein; S-segment: N, nucleoprotein; NSs, non-structural protein.

The newly discovered SBV has sequence similarities to other viruses in the *Peribunyaviridae* family, genus *Orthobunyavirus*, such as Akabane, Aino and Shamonda. Further sequence analysis also revealed high similarities with Sathuperi, and Douglas viruses [2]. SBV is an enveloped, single-stranded, negative-sense RNA virus with three genomic segments: L (large), M (medium) and S (small) segments (Figure 1A) [3]. The RNA-dependent RNA polymerase (RdRp) is encoded by the L segment, whereas the M segment encodes a polyprotein that is further cleaved into the envelope glycoproteins Gn and Gc and the non-structural protein NSm (Figure 1B). Overlapping open reading frames (ORFs) of the S segment encode the nucleoprotein N and the non-structural protein NSs (Figure 1B) [1,4]. Like other viruses in the order *Bunyavirales*, the NSs of SBV has been shown to be a major virulence factor that downregulates host-cell mRNA synthesis and type I interferon production in mammalian cells, thereby enhancing viral replication [4,5]. SBV is transmitted by culicoides midges [1]. 

## 2. Epidemiology

Various virulent arboviruses, such as West Nile virus, Rift Valley fever virus, Chikungunya virus, Zika virus, and Bluetongue virus (BTV), have emerged and caused epidemics in humans and/or animals in North and South America, the Arabian Peninsula or Europe. Their emergence has been partially attributed to the phenomenon of climate change [6]. In the fall of 2011, Schmallenberg virus appeared in Northern Europe, where also BTV serotype 8 (BTV-8) first appeared in 2006 [1,7]. Schmallenberg virus infection manifested itself as congenital defects in newborn calves, lambs and kids, as well as an arthrogryposis and hydranencephaly syndrome [8]. SBV quickly spread to the rest of the continent, with wind playing an important role in the transmission of the virus, as infected midges are easily carried on air currents [9]. The rate of spread of SBV is estimated to be in the range of 0.9 to 1.5 km/day [10]. Despite a high seroprevalence of up to 98% reported in outbreak regions in 2011 and 2012 [11], new infections occurred again in the summer of 2012 in the same regions. Both serological and genome detection methods revealed the re-emergence of SBV infection in Belgium in 2012 [12]. It became evident that the cold winter season could not eradicate the virus since new cases were also observed in ruminants in Germany in June 2012 [13]. In late 2014, SBV outbreaks in cattle and sheep were once again detected in The Netherlands; and in 2016, an increase in SBV-specific antibody titers and prevalence in heifers was noted in The Netherlands [14]. Additionally, the detection of SBV genomic sequences in aborted calves in Belgium in 2015 was a further indication of SBV circulation in the area at the time [15]. Although there were several years of limited to no circulation of the virus in the UK and France, outbreaks of the disease occurred again in 2016 and 2017 [16,17].

The original source of SBV is still unclear; however, there are reports of SBV cross-reactive antibodies to other Simbu serogroup viruses found in African cattle, prior to and after the initial European outbreak [18,19,20]. A report from Jordan in 2013 indicated detection of antibodies against Aino virus, another Simbu serogroup virus, in ruminants on farms where similar clinical symptoms associated with SBV infections were observed [21]. Turkey reported detecting SBV RNA in aborted cattle and sheep fetuses a year after the initial outbreak in Europe, suggesting a spread of the virus from Northern Europe [22]. Furthermore, blood samples collected before 2011 were found to be positive for SBV antibodies by ELISA [23]. Since the SBV ELISA has low specificity, the possibility of potentially detecting cross-reactive antibodies induced by other Simbu serogroup viruses cannot be ruled out. Remarkably, there have been multiple reports of fetal malformation in ruminants from the Mediterranean region, suggesting a possible circulation of Simbu serogroup viruses in this region [21,22,23,24].

Insect vectors such as mosquitoes and biting flies (*Culicoides* spp.) are often responsible for the transmission of bunyaviruses associated with human and animal diseases in Asia and Africa. Immediately after the discovery of SBV, it became evident that *Culicoides spp*. play a role in its transmission [25]. SBV genomic sequences were detected in biting flies, namely the *Culicoides obsoletus* species group of the *Ceratopogonidae* family, and it was shown that multiple *Culicoides spp.* (*Culicoides dewulfi*, *Culicoides chiopterus*, *Culicoides punctatus*, etc) were positive for SBV genomic markers. Culicoides caught as early as summer and autumn of 2011 in Belgium [26], Italy [27], The Netherlands [28], and Denmark [25] were found positive for SBV genomic markers. Interestingly, *Culicoides sonorensis,* a vector for BTV, was also shown to support the replication and dissemination of SBV under laboratory conditions [29]. The question of how the vector-borne SBV persists over winter has not been solved yet; however, one field study has demonstrated that the potential mechanism is transovarial transmission in the *Culicoides* vector [30].

Vertical transmission of SBV from infected dam to fetus occurs during the first and early-second trimester of gestation and results in abortion, stillbirth and birth of malformed newborns [31,32]. Although experimentally infected animals shed SBV RNA in feces, oral and nasal fluids [33], direct transmission of SBV from infected ruminants to naïve animals by contact or oro-nasal/feco-oral routes has not been reported [33]. Both, oral inoculation of cattle and nasal inoculation of sheep failed to produce viremia in the animals [33]. Interestingly, SBV was detected in semen from infected bulls [34]; however, transmission of SBV from infected bulls to dams either through natural mating or artificial insemination has not been extensively studied yet [34]. In one study, viral RNA was isolated from blood samples of cattle experimentally injected with SBV-RNA-positive semen [34]. The presence of SBV RNA in amniotic fluid and fetal tissues [35,36] was suggested in a previous review on *Orthobunyaviruses* as one possibility the virus may persist over winter [37].

Multiple domestic and wild animal species have been shown to be susceptible to SBV infection under natural and experimental conditions. This was determined through direct and/or indirect detection of SBV in animals with clinical manifestations or subclinical infections [14]. Importantly, overt clinical manifestations of SBV infection have been seen exclusively in domestic ruminants [14], whereas only indirect serological evidence of SBV infection has been reported for wild ruminants (e.g., alpaca, buffalo, deer, chamois, mouflon, bison), zoo animals (e.g., kudu, zebra, oryx), and some other mammalian species (e.g., horse, wild boar) [38,39,40,41,42]. Interestingly, virological and serological evidence of SBV infection has been also reported in dogs [43]. Experimental infection of piglets with SBV resulted only in seroconversion, and no RT-PCR positivity was detected, suggesting the inability of the virus to efficiently replicate in this host species [44]. 

## 3. Clinical and Pathological Findings

Infection of adult ruminants with SBV usually results in non-specific clinical signs. In cattle, SBV infection often manifests as a mild and transient disease, with anorexia, hyperthermia, and in some animals with diarrhea and reduced milk yield (up to 50%) [1,45]. In contrast, SBV infection in adult sheep and goats is mostly subclinical. Acute clinical cases of SBV are not common, but there are a few reports of clinical disease in adult animals (6% cattle, 3% sheep and 1% goats) [46]. Clinical signs of diarrhea and reduced milk yield have been reported at least once in goats [47]. Even though the causal relationship has not been clearly established, there have been reports of fever, diarrhea and reduced milk yield in sheep [46]. Under experimental conditions, in sheep and cattle, SBV infection exhibits a short viremic period of 5–7 days, which starts at day 2 or 3 post infection (pi) and peaks around day 4 pi [47]. The clinical outcomes of abortion, stillbirth, and malformed newborns associated with SBV infection in cattle and sheep are similar to those observed for other Simbu serogroup viruses such as Akabane and Aino [8,48]. A relationship between herd immunity and the birth of congenitally malformed newborns has been observed during SBV infection. A decline in herd immunity is followed by an increase in seroprevalence against SBV [45,49]. Common musculoskeletal deformities observed in fetuses during transplacental infection include arthrogryposis, lordosis, scoliosis, torticollis and brachygnathia inferior [37,50]. Sacral spina bifida and cleft palate are also observed in stillborn lambs. Meanwhile, musculoskeletal defects are not uniform in the case of twin gestation, in which case, one twin may present malformations, whereas the other is born healthy without any malformation or clinical signs [37]. A recent study revealed that experimental *in utero* infection of bovine fetuses resulted in a very low incidence rate of fetal abortion/malformation; only one abortion and one malformed fetus out of 36 experimentally *in utero* SBV-infected pregnant heifers were reported when the animals were infected between 60 and 150 days of gestation (first and second trimester) [51].

At necropsy, hydranencephaly, porencephaly, lissencephaly, hydrocephalus, cerebellar and cerebral hypoplasia and micromyelia are commonly observed in the central nervous system (CNS) of SBV-infected young ruminants. Among the microscopic lesions, glial nodules mainly in the mesencephalon and hippocampus of lambs and goats, lymphohistiocytic meningoencephalomyelitis, as well as neuronal degeneration and necrosis mainly in the brain stem of calves are observed [52]. The musculoskeletal defects manifested as arthrogryposis in fetuses are most likely due to lesions in the spinal cord [3]. Hence, these musculoskeletal lesions in SBV-infected aborted fetuses or neonates led to the description of the arthrogryposis and hydranencephaly syndrome (AG-HE syndrome) [14].

## 4. Immunity

Infections of Schmallenberg virus in naïve populations spread quickly but result in solid protective immunity. This was evident when, within a two-month period, many cattle herds in Germany became infected [53]. Studies indicate that in the majority of SBV-infected cattle and sheep, anti-SBV antibodies last for at least 38 and 48 months, respectively [14]. A recent report indicates that among 17 naturally infected cattle studied over a period of 6 years, three animals became seronegative, while the other 14 animals still had measurable anti-SBV antibodies after six years [53]. Most calves and lambs born to SBV-infected cows or ewes were shown to be protected from SBV infection for at least the first six and four months, respectively [53,54]. Under experimental conditions, anti-SBV antibodies are detected between 14 and 21 days post-infection in cows and sheep [33,45,49]. Interestingly, the role of CD8+ T lymphocytes seems insignificant during SBV infection as these cells are not detected after initial infection, implying that virus clearance is most likely independent of T-cell-mediated cytotoxicity. However, CD8+ T-cells may be involved in the protective immune response against the virus during secondary exposures. In addition, between 3–7 days post-infection, the number of CD4+ T helper cells decrease and SBV genomic markers are not detectable in peripheral blood leukocytes [33].

## 5. Diagnosis

In adult animals, SBV mostly causes subclinical infections; however, occasionally, especially in cattle, clinical signs of fever, diarrhea and a reduction in milk yield are not uncommon [1]. In transplacental infections, congenital CNS and musculoskeletal malformations, such as the AG-HE syndrome, aplasia or hypoplasia of the cerebrum or cerebellum and hydranencephaly are observed [33,45,49]. Virological and/or serological diagnosis is needed to confirm suspicion of SBV infection because of similarities between clinical features of SBV and other ruminant virus infections [33].

SBV can be isolated in multiple insect and mammalian cell types such as: BHK-21 (baby hamster kidney), Vero (African green monkey) and KC (*Culicoides sonorensis*) cells [1]. Only mammalian cells are permissive to SBV infection with cytopathic effects. However, virus isolation is not always possible from clinical specimens including blood samples, due to the low viral load in most samples. Real-time reverse transcription quantitative polymerase chain reaction (RT-qPCR) targeting the L and S segments of the SBV genome allows sensitive and specific detection of SBV RNA, which is indicative of SBV infection [55]. The presence of SBV RNA in various organs has been tested by RT-qPCR, but only some of them were determined as positive [31,56]. SBV genomic sequences are readily detected in the cerebrum, spinal cord, placental fluid and umbilical cord of malformed lambs or calves [31]. The brain stem is also an appropriate sample for the detection of SBV RNA [57].

Detecting anti-SBV antibodies in serum represents an indirect method for the diagnosis of SBV infections. Both, the virus neutralization test (VNT) and enzyme-linked immunosorbent assay (ELISA) have been developed as techniques for serological diagnosis. The former method is time-consuming; hence its use is restricted as a confirmatory test. On the other hand, the ELISA is a rapid, less expensive and high-throughput test. However, interpretation of test results in areas where other Simbu serogroup viruses are circulating could give false-positive results due to potential antibody cross-reactivity and, therefore, caution should be exercised in interpreting these results [58]. However, a unique advantage of the ELISA test is that it can be used to detect anti-SBV antibodies in milk [59]. On the other hand, discrepancies in specificity and sensitivity between VNT and ELISA have been reported [60]. ELISA tests seem to exhibit lower specificity and sensitivity than VNTs [60]. Anti-SBV antibodies can also be detected by indirect immunofluorescence assay [61]; however, this method is not often used as a routine detection technique. Immunohistochemistry and *in situ* hybridization are techniques which are employed for the detection of SBV proteins and genomic RNA in paraffin-embedded tissue sections, respectively [62].

## 6. Surveillance and Vaccination

Surveillance of the dynamics of competent vectors and vector infection rates seems to be the optimal strategy for predicting future SBV outbreaks. Furthermore, vaccination of replacement stocks and control of insect populations are the two most important methods for the prevention of SBV outbreaks [14], as vaccination in particular helps reduce SBV infection in ruminants [61]. Farmers in some countries, however, are unwilling to vaccinate their animals against SBV, claiming vector surveillance to be a more effective prevention strategy than vaccination. The re-emergence of SBV in Germany and The Netherlands in late 2014, and more recently in Belgium, France and the UK, is an indication that SBV is able to infect and disseminate in cattle and sheep flocks in the face of declining immunity [17,63,64]. SBV re-emerging events should be a reminder for farmers to regularly implement SBV vaccination of their animals.

## 7. Inactivated Vaccines

Inactivated SBV vaccines have been developed shortly after the initial isolation and characterization of the virus in Europe by various companies [65,66]. These vaccines effectively prevent viremia and clinical disease, including the prevention of fetal malformation and premature birth or stillbirth [67,68]. Three commercial vaccines, namely Zulvac SBV (Zoetis), Bovilis SBV (MSD Animal Health) and SBVvax (Merial) have been in use in Europe to protect sheep and cattle from SBV infection (see Table 1). In an experimental study using cattle and sheep, the onset of inactivated vaccine-induced immunity was demonstrated as early as 2 weeks after vaccination [65,66]. Furthermore, the efficacy of these vaccines appeared to be dependent on the production cell line and the virus titer in the vaccine [66].

## 8. Modified-Live and Subunit Vaccines

Recombinant modified-live vaccines with NSm and/or NSs deletions in the M and S segments of SBV have been developed and conferred a high level of protection from infection [69]. Although these vaccines are DIVA (differentiate infected from vaccinated animals) compatible, safety concerns related to reversion to virulence may prevent their wider use. It was reported that serial passages of a NSs-deleted, attenuated virus in cell culture was able to restore its virulence when a single mutation was acquired in the Gc protein [70]. Using baculovirus produced Gc or Gc and Gn as a subunit vaccine generated a low level of neutralizing antibody response but did not protect against SBV challenge [71]. Another subunit vaccine based on the SBV Gc amino terminal domain (aa. 468–702), expressed in human embryonic kidney (HEK) cells; however, provided partial protection in cattle (three out of four animals were protected after challenge) [72]. Meanwhile, another subunit vaccine construct, which contained the entire ectodomains of Gc and Gn in a covalently linked fashion, conferred only low protective efficacy in cattle (one out of four animals protected after challenge) [72]. In contrast, full protection was obtained when an antigen containing the covalently linked Gc amino-terminal domains of both SBV and Akabane viruses was used [72]. Meanwhile, a DNA vaccine encoding the SBV Gc amino-terminal domain and the SBV nucleoprotein reduced viremia and protected animals against weight loss [73]. Vaccination with recombinant Equine Herpes Virus 1 or Modified Vaccinia Virus Ankara expressing the SBV Gc amino-terminal domain resulted in partial (two out of four animals) or full protection in cattle, respectively [74] (see Table 1). In general, in order to prevent fetal infection with SBV, vaccination should be targeted to breeding animals.

## 9. Potential for Re-Emergence of SBV

Like other viruses in the Simbu serogroup such as Akabane and Aino, SBV re-emerges when herd immunity declines and favorable conditions for the vector population occur [37]. Aino and Akabane virus-based epidemics occur every 3–6 years in Japan, as more naïve animals become available [50,76]. Similarly, in Australia, Akabane virus outbreaks occur every 10–15 years, due to the temporary change in the vector populations and the availability of naïve susceptible animals [77]. In 2015, the re-emergence of another arbovirus, BTV serotype 8, which shares the same vector species as SBV, occurred in Europe after several years of absence of clinical disease [78]. The fact that only 20% of newborn animals carried anti-SBV antibodies in 188 herds examined during a cross-sectional study in Belgium in 2012, demonstrates a significant loss of herd immunity compared to 2011 [79]. The mean herd seroprevalence in calves declined from 65.7% and to 20.6% [79], which represents a recipe for another SBV outbreak in this country. Between 2014 and 2015, no anti-SBV antibodies were detected in ruminants in the UK and Ireland, suggesting an absence of SBV circulation in the ruminant and vector population in these countries [80,81]. In contrast, from 2016 to 2018, virus circulation at low levels was detected in many countries in Europe, such as Belgium [15], UK [17] and France [16]. These epidemiological data re-enforce the idea of a possible resurgence of SBV infections when favorable conditions for its occurrence such as high proportion of naïve susceptible animals and increased vector populations exist [82].

## 10. Conclusions

Schmallenberg virus, a newly emerged arbovirus, was first reported in Central and Northwestern Europe in the fall of 2011. The initial source of introduction has not been identified to date. In addition, the mechanism by which the virus is maintained during the winter period when the vector population is low or absent remains unknown. Therefore, future studies should address the possibility of SBV introduction/re-introduction through infected midges imported from tropical or endemic regions where serological, molecular and vector prevalence of SBV has been reported. It is equally important to examine whether persistently infected, clinically healthy offspring born to infected domestic and wild ruminants exist and act as a constant source of SBV infection. Additional investigations of the competence of other arthropod vectors in the transmission of SBV, as well as for other viruses of the Simbu serogroup, are needed. Furthermore, studies on the cross-reactivity of current diagnostic tests and cross-protection between viruses from the Simbu serogroup need to be conducted. The major lesson learned from the emergence of SBV, is that maintaining vigilance and expertise is of great importance to be able to respond rapidly to new threats to animal and public health.

## Figures and Tables

**Table 1:** Summary of SBV Vaccines and SBV Vaccine Candidates.

Type of Vaccine	Host Species Evaluated			DIVA Compatibility	References
	Mice	Cattle	Sheep		
**Inactivated vaccines:**					
Binary ethylenimine inactivated	NO	YES	YES	NO	[65,66]
Bovilis SBV (MSD Animal Health)	NO	YES	YES	NO	[67]
Zulvac SBV (Zoetis)	NO	YES	YES	NO	[75]
SBVvax (Merial)	NO	YES	YES	NO	[68]
**Genetically modified live virus vaccines:**					
Recombinant NSm and/or NSs deletion mutants	YES	YES	YES	YES	[69]
**DNA vaccines:**					
SBV Gc (N-terminal)	YES	NO	NO	YES	[72]
SBV Nucleoprotein	YES	NO	NO	YES	[73]
SBV Gn (ectodomain), SBV Gc (ectodomain 1 and 2)	YES	NO	NO	YES	[73]
**Virus-vectored vaccines:**					
Recombinant Equine Herpes Virus 1, Gc (N-terminal)	NO	YES	NO	YES	[74]
Modified Vaccinia Virus Ankara, Gc (N-terminal)	YES	NO	YES	YES	[74]
**Recombinant subunit Vaccines:**					
Baculovirus-expressed Gc or Gc/Gn	NO	YES	NO	YES	[71]
Gc (N-terminal), HEK cells	YES	YES	NO	YES	[72]
Gc + Gn linked ectodomains, HEK cells	YES	YES	NO	YES	[72]
Gc (N-terminal) of SBV and Akabane, HEK cells	YES	YES	NO	YES	[72]
